# Social defeat models in animal science: What we have learned from rodent models

**DOI:** 10.1111/asj.12809

**Published:** 2017-04-24

**Authors:** Atsushi Toyoda

**Affiliations:** ^1^ College of Agriculture Ibaraki University Ami Ibaraki Japan; ^2^ Ibaraki University Cooperation between Agriculture and Medical Science (IUCAM) Ami Ibaraki Japan; ^3^ United Graduate School of Agricultural Science Tokyo University of Agriculture and Technology Fuchu‐city Tokyo Japan

**Keywords:** depression, feed, metabolome, social defeat

## Abstract

Studies on stress and its impacts on animals are very important in many fields of science, including animal science, because various stresses influence animal production and animal welfare. In particular, the social stresses within animal groups have profound impact on animals, with the potential to induce abnormal behaviors and health problems. In humans, social stress induces several health problems, including psychiatric disorders. In animal stress models, social defeat models are well characterized and used in various research fields, particularly in studies concerning mental disorders. Recently, we have focused on behavior, nutrition and metabolism in rodent models of social defeat to elucidate how social stresses affect animals. In this review, recent significant progress in studies related to animal social defeat models are described. In the field of animal science, these stress models may contribute to advances in the development of functional foods and in the management of animal welfare.

## Introduction

Although animals and humans are continually affected by environmental stresses, such as heat, infection and aggression, they generally maintain a homeostatic balance via their endogenous strength, namely, resilience (Selye 1956). Unfortunately, strong and chronic stresses have marked effects on animal bodies and can induce various disorders. Abnormal behaviors are often observed under conditions of severe stress. In humans, several mental disorders are induced by stress, and major depression is a particularly common disease with a lifetime prevalence of 2%–20% worldwide (Weissman *et al*. 1996). Furthermore, stress management in animals, such as livestock, experimental animals and zoo animals, has recently become a more important issue, compared with over the past few decades, because of the increased worldwide focus on animal welfare (Grandin & Shivley 2015). Therefore, the various aspects of studies on animal stresses warrant investigation.

Social conflicts frequently occur in animal and human societies, and unavoidable stresses can worsen the quality of life in both animals and humans. In our research group, rat and mouse models of social defeat have been developed and characterized in order to elucidate the mechanisms of psychiatric disorders such as depression. Moreover, rodent models of social defeat are applicable to group‐housed animals on farms and in zoos, because social interactions among conspecific animals often induce serious stress, particularly in inferior animals. Therefore, the rodent models of social defeat can potentially provide significant and basic information for understanding several of the physiological and behavioral abnormalities induced by social stress between individuals.

Various animal models of depression were developed and widely used in depression studies (Nestler & Hyman 2010) (Table 1). Forced swimming and tail suspension tests, for example, are popular for screening antidepressants; however, there are serious concerns regarding the predictive validity of such acute stress models (Cryan & Holmes 2005). Acute administration of antidepressants can cure the ‘depressive behavior’ in these models, although only chronic administration of antidepressants can alleviate the depressive symptoms in human patients. Among the depression models, the chronic unpredictable mild stress model (Willner 2005) and the chronic social defeat stress (CSDS) model (Miczek 1979) are more reasonable for depression studies compared to other acute stress models from the points of view of several validities (Cryan & Holmes 2005; Nestler & Hyman 2010). In particular, the mouse models of CSDS, which have been comprehensively studied using omics techniques, have produced significant knowledge about depression (Krishnan *et al*. 2007). Our research group has focused on deficits of behavior, nutrition and metabolism in CSDS models, and in this review recent progress in the use of CSDS models is described.

**Table 1:** Representative depression models of rodents

Model	Stress type	Reference
Forced swim	Acute, physical, emotional	Porsolt *et al*. (1977)
Tail suspension	Acute, physical, emotional	Steru *et al*. (1985)
Mild stress	Chronic, physical, emotional	Willner (2005)
Maternal deprivation	Chronic, emotional	Schmidt *et al*. (2002)
Social defeat	Chronic, physical, emotional	Golden *et al*. (2011)
Subchronic social defeat	Chronic, physical, emotional	Goto *et al*. (2014)
Witness stress	Chronic, emotional	Sial *et al*. (2016)
LPS injection	Acute, chemical	Shen *et al*. (1999)

LPS, lipopolysaccharide

## Animal Social Defeat Models

**Figure 1:**
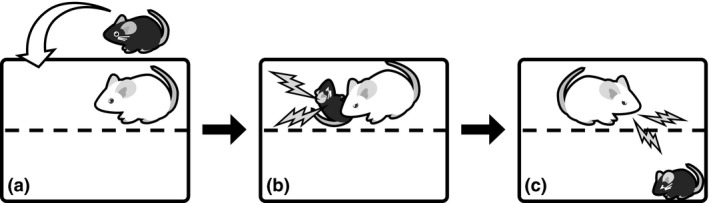
Development of a mouse model of social defeat. An ICR mouse is housed in a compartment separated by a transparent acrylic divider containing many holes (a). After a few days of habituation, a C57BL/6J (B6) mouse is introduced into this compartment, and normally the ICR mouse will severely attack the B6 mouse (b). After several minutes of this social conflict, the B6 mouse is moved to an adjacent compartment for the remainder of the day (c). The following day, the B6 mouse is subjected to social conflict with another ICR mouse. This sequence of physical and psychological stress is repeated for 10 days to induce depressive symptoms in the B6 mouse.

Animals subjected to CSDS are good models for gaining an understanding of psychiatric disorders and abnormal behaviors in animals. Previously, rat (Miczek 1979), mouse (Krishnan *et al*. 2007) and hamster (Potegal *et al*. 1993) models of CSDS have been reported and extensively studied. Furthermore, pig (Ruis *et al*. 2001), zebra fish (Oliveira *et al*. 2016), *Drosophila* (Penn *et al*. 2010) and cricket (Rillich & Stevenson 2014) models of defeat have been developed and characterized. Figure 1 shows a popular paradigm for production of mouse models of social defeat, using ICR mice as the resident and C57Bl/6J (B6) mice as the intruder (Golden *et al*. 2011; Goto & Toyoda 2015a). Aggressive ICR mice, which are screened before conducting the paradigm, are housed for a few days in a compartment of the home cage divided by a transparent acrylic board, which contains small holes that allow the circulation of odors, pheromones and vocalizations. Thereafter, a B6 mouse is introduced into the territory of an ICR mouse and the ICR mouse typically attacks the B6 mouse, which is characterized by chasing, grooming, and biting (physical stress). After a short duration of aggression, the B6 is moved to a neighbor compartment of the ICR and kept for the remainder of the day (psychological stress). The following day, the B6 mouse is subjected to another ICR mouse in the same manner. In the normal protocol, B6 mice are subjected to 10 daily sessions of physical stress and psychological stress, as described above, and these B6 mice become CSDS models that display various depressive‐like features.

Although different versions of the paradigm for developing CSDS models are used for various purposes, it is generally not possible to prevent injuries to B6 subordinates from physical contact. Therefore, our protocol for the paradigm was modified and the duration of physical contact was reduced from 5 min to 30 s each day to minimize the severe injuries derived from biting by ICR mice (Goto & Toyoda 2015a). Our social defeat paradigm that involves less physical stress is considered to be appropriate for developing mouse models of human depression, because psychosocial and emotional stresses mainly impact human mood and precipitate mental disorders. Actually, our chronic social defeat model of mice showed several depressive symptoms as described below. Recently, a mouse model of defeat has been developed in which the focal mouse is made to witness stress (Sial *et al*. 2016). In this paradigm, only mental stress is induced by exposing a B6 mouse to conflict between ICR and B6 mice in an adjacent room. In this paradigm, therefore, the subordinate mouse does not suffer physical stress or wounding. As in the conventional mouse models of social defeat, various depressive symptoms, such as weight loss, decreased social behavior and anxiety, develop in mice that witness stress (Sial *et al*. 2016). Because chronic but not acute fluoxetine administration is effective in alleviating the depressive symptoms in model mice, the mice that witness stress satisfy the prediction validity of a depression model. Moreover, it has been reported that a BALB/c mouse model of social defeat shows greater vulnerability to stress than does the B6 mouse model (Savignac *et al*. 2011).

Normally, different strains of rodents are used as resident and intruder for the production of models of social defeat. Previously, we attempted to develop a CSDS model using Wistar rats as both resident and intruder; however, it was difficult to induce constant aggressive behaviors in resident Wistar rats. Therefore, we maintained an aggressive colony of Wistar rats as the residents in our laboratory and produced a Wistar rat model of chronic social defeat (Iio *et al*. 2011). For the CSDS paradigm in both mice and rats, the aggressive behaviors of residents are considered to be critical to achieve defeat model reproducibly. Since it has previously been described that aggressive ICR mice constantly attack B6 mice in less than 50% of encounters (Golden *et al*. 2011), it is important to conduct selection of aggressive residents before performing the CSDS paradigm, in order to produce a successful CSDS model. Recently, variations of the social defeat model, including a juvenile model and a female model have been reported (Iñiguez *et al*. 2014; Solomon 2017), and analysis of these models will enable us to gain a better understanding of stress‐induced abnormal behaviors and psychiatric disorders such as depression.

## Behavioral Analysis

Mouse and rat models of social defeat have been widely characterized by various experimental techniques. In this section, the behavioral features of the models, particularly those that are important in animal science, are described.

### Eating behavior

Some eating disorders, including anorexia and hyperphagia, are frequently observed in depression (American Psychiatric Association 2013), and these symptoms are also observed in depression models. In the Wistar rat model of social defeat developed in our laboratory, the food intake and body weight gain of this rat model were suppressed compared to control rats (Iio *et al*. 2011, 2012a). These results are consistent with those obtained for another rat model of defeat (Rygula *et al*. 2005). Therefore, we focused on analyzing the mechanisms whereby food intake is suppressed in the rat model of social defeat (Iio *et al*. 2012a, 2014).

Although decreases in blood glucose level and leptin gene expression in adipose tissue were observed in the model rats (Iio *et al*. 2014), the level of malonyl‐coenzyme A (CoA), a feeding regulator in the hypothalamus, was elevated (Iio *et al*. 2012a). Normally, the level of malonyl‐CoA in the hypothalamus is increased by leptin and blood glucose, which suppresses eating behaviors via altered hypothalamic expression of several neuropeptides related to eating behaviors (Lane *et al*. 2008). Considering the peripheral metabolism of the rat social defeat model, it was assumed that the level of hypothalamic malonyl‐CoA would be decreased. However, despite the low levels of peripheral leptin and blood glucose in the defeat model, the level of hypothalamic malonyl‐CoA was abnormally increased to induce anorexia (Iio *et al*. 2012a). Furthermore, signals downstream of hypothalamic leptin in the rat social defeat model appeared to be normal (Iio *et al*. 2014). Consequently, CSDS may perturb the regulation of hypothalamic malonyl‐CoA. In addition, anorexia in depressive patients may be linked to hypothalamic malonyl‐CoA. Hence, by targeting malonyl‐CoA and its related enzymes in the hypothalamus, it may be possible to develop novel therapeutic drugs for anorexia.

Eating behaviors have also been investigated in the mouse model of chronic social defeat. Krishnan *et al*. (2007) reported that CSDS decreases food intake and body weight in B6 mice. In contrast, Goto *et al*. (2014) described that CSDS does not decrease food intake, but instead increases body weight gain following polydipsia‐like features. Because body water content also increases in CSDS mice compared to control mice, increased body weight gain may be attributable to polydipsia (Goto *et al*. 2014). Differences in the food intake and body weight of CSDS mice are considered to be dependent on factors such as the experimental environment, breeder and feed, although the details are unknown. The food intake and body weight of CSDS mice may be influenced by the strength of physical contact with resident ICR mice. Indeed, a previous report has described that 10 days of CSDS (10 min/day) does not change body weight, but that 10 days of CSDS during which the intruder suffers only one defeat daily increases body weight compared to control mice (Savignac *et al*. 2011). As mentioned above, in order to reduce physical stress, such as that resulting from injuries inflicted by ICR mice, the protocol of the subchronic and mild social defeat stress (sCSDS) model has a reduced contact time with ICR compared to the standard protocol (Golden *et al*. 2011); therefore, sCSDS animals may not experience the severe stress that decreases food intake and body weight (Goto *et al*. 2014). As increases in food consumption and body weight gain are observed in depressive patients (American Psychiatric Association 2013), sCSDS mice will be a good model of depression associated with such symptoms. However, there may not be a simple correlation between the intensity of physical stress and food consumption and body weight gain, because a previous report has described that witnessing stress can also decrease body weight (Sial *et al*. 2016). A detailed analysis of this issue should therefore be carried out in the future. Furthermore, social defeat models of livestock should be developed and intensively investigated, because there is little information about the effects of social defeat stress on feeding and growth. Neuropeptides related to eating behaviors are known to be linked to depression (Lutter & Nestler 2009), and analysis of feeding behavior and related neuropeptides in various stress models may provide important insights into eating disorders and deficient body weight control.

### Depression‐like behavior

**Figure 2:**
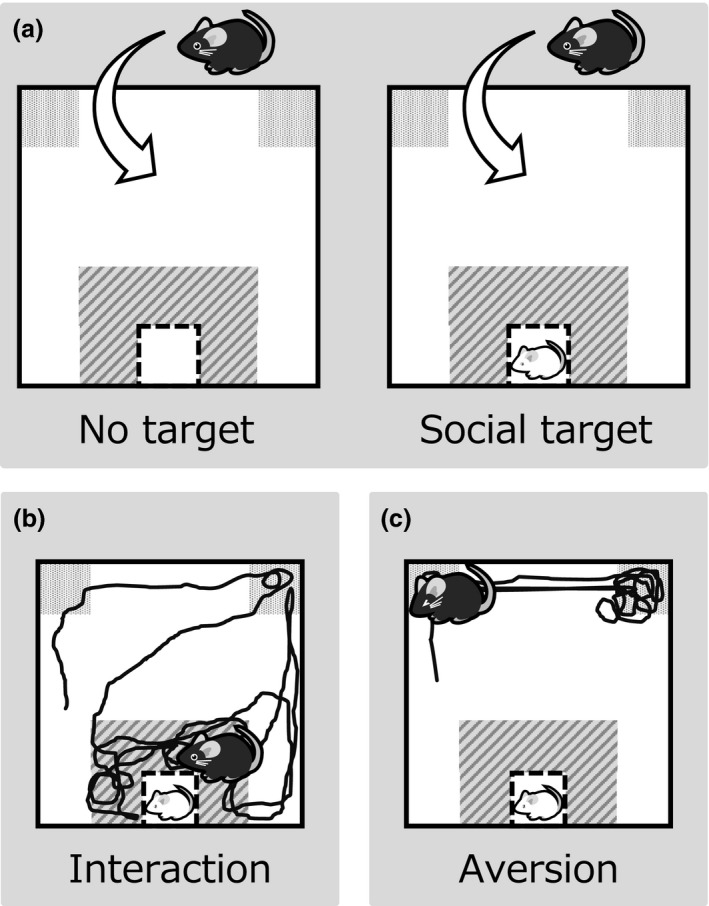
Social interaction test for evaluation of defeated mice. The social behavior of stressed and control B6 mice is monitored with or without ICR mice (a). Control and resilient mice engage in social interaction with ICR mice (b), whereas susceptible mice avoid ICR mice (c).

It is difficult to evaluate depressive behaviors objectively in mice and rats. What is the behavior associated with the face validity of the depression model? The forced swimming test (FST) and tail suspension test (TST) have been used to evaluate depression‐like behaviors (Porsolt *et al*. 1977; Steru *et al*. 1985). Drugs showing antidepressant effects have been widely screened using the FST and TST, because some antidepressants can shorten the immobile behavior in these tests. Unfortunately, acute administration of antidepressants can reduce the immobility time in FST and TST, which is not consistent with clinical situations (Cryan & Mombereau 2004). In general, antidepressants are effective for depressive patients in chronic administration, but not acute administration. Therefore, drug screening using the FST and TST is not based on predictive validity for depression. Moreover, the immobility time of the FST was extended in CSDS rats (Iio *et al*. 2011), but the immobility time of the FST and TST was not changed in sCSDS mice (Goto *et al*. 2014). In the case of bipolar disorder model mice, it has been reported that the immobility time of the FST was unexpectedly reduced (Kasahara *et al*. 2016). Collectively, the depression‐like symptoms of mice and rats cannot be completely reflected in evaluation tests of despair such as the FST and TST. Accordingly, social interaction tests have been used to evaluate depression‐like behaviors in social defeat models (Fig. 2a) (Golden *et al*. 2011; Goto *et al*. 2014; Goto & Toyoda 2015a). As shown in Figure 2a, a B6 mouse was introduced into an open field arena with or without an ICR mouse in a wire mesh box. The duration for which the B6 mouse remains in the interaction and corner zones are measured. Since normal and healthy B6 mice are motivated to explore, the B6 mouse approaches the ICR mouse (Fig. 2b). However, a B6 mouse subjected to CSDS avoids the ICR mouse (Fig. 2c), although, interestingly, some populations of CSDS mice show stress resilience characteristics (Krishnan *et al*. 2007).

Various studies focusing on the difference between resilience and susceptibility have been carried out on the central nervous system (Krishnan *et al*. 2007; Golden *et al*. 2013; Bagot *et al*. 2015) and the immune system (Hodes *et al*. 2014), because the underlying mechanism of this stress vulnerability is likely to be linked to the pathology of depression. Recently, we revealed that resilience to social defeat stress is affected by diet (Goto *et al*. 2016a). Namely, mice fed a non‐purified diet (MF) are more resilient than those fed a semi‐purified diet (AIN‐93G), although the mechanisms underlying this difference are not known. In future studies, the dietary substances that enhance stress resilience should be identified. Indeed, Yao *et al*. (2016) reported that the sulforaphane and glucoraphane contained in broccoli enhance resilience to social defeat stress. Therefore, it is assumed that various natural resources and foods, including plants and fruits, could increase stress resilience, and in the future, it will be fruitful to explore candidates for the medication of depression using social defeat models.

## Metabolic Features of Rodent Models of Social Defeat

As described above, social defeat models have been analyzed using various biochemical and molecular biological methods, including comprehensive methods such as DNA microarrays (Krishnan *et al*. 2007). Recently, using metabolomics, we attempted to identify the metabolic pathways influenced by CSDS. Taurine levels in the liver and plasma of CSDS rats were shown to be higher than those in control rats (W. Iio *et al*., unpublished data). Similarly, a clinical report has described that blood taurine concentrations in depressed patients are higher than those of controls (Altamura *et al*. 1995). Although details are unknown, depressive symptoms may be linked to blood and tissue taurine. Therefore, we focused on the effect of taurine on behavior and the central nervous system. Iio *et al*. (2012b) reported that taurine supplementation for 4 weeks had antidepressant‐like effects because chronic taurine decreases the immobility time of the FST. Furthermore, Murakami and Furuse (2010) reported that chronic taurine administration produced antidepressant‐like effects in mice. We therefore attempted to elucidate the hippocampal signal cascades that play a key role in depression in rats fed taurine (Iio *et al*. 2012b). Taurine supplementation for 2 weeks (45 mmol/kg diet) did not affect behavior in the FST; however, AKT, GSK3b, ERK, and CREB were phosphorylated. Finally, CaMKII was phosphorylated for 4 weeks concomitant with taurine feeding, suggesting that phosphorylation of CaMKII is critical in the antidepressant‐like action of taurine (Iio *et al*. 2012b).

Metabolome studies were conducted in the plasma, liver and cecum digesta of sCSDS mice (Goto *et al*. 2015b; Aoki‐Yoshida *et al*. 2016). Although no significant change was observed in the plasma metabolome of model mice, metabolites such as taurocyamine (GES), phosphorylcholine, alanyl‐alanine, and 1‐methylnicotinamide, in the liver were increased by sCSDS (Goto *et al*. 2015b). GES is a metabolite located downstream of taurine, and thus social defeat stress may have a general effect on taurine metabolism in the liver.

Social defeat stress causes brain dysfunction following inflammation in the peripheral tissues and central nervous system (McKim *et al*. 2016). Although the mechanisms whereby liver taurine and GES levels are increased by social defeat stress are unclear, the anti‐inflammatory actions of taurine and its derivatives may be important for liver function under stressful conditions (Marcinkiewicz & Kontny 2014). Inflammation is, nevertheless, profoundly related to mental illness (Mechawar & Savitz 2016), and therefore future studies are needed to determine whether taurine supplementation would be effective in alleviating mental illness‐related inflammation. Increases in cholic acid and decreases in 5‐aminovaleric acid (5‐AV) have been observed in the cecal contents of sCSDS mice (Aoki‐Yoshida *et al*. 2016). Since cholic acid is an antagonist for *N*‐methyl‐d‐aspartate (NMDA) and γ‐aminobutyric acid (GABA) receptors (Schubring *et al*. 2012), increased cholic acid in the cecum digesta may affect the peripheral and central nervous systems. In addition, 5‐AV modifies GABA neurons via various mechanisms (Nanavati & Silverman 1989; Fritsche *et al*. 1999), including inhibition of GABA uptake (Tasaka *et al*. 1989), inactivation of GABA aminotransferase (Nanavati & Silverman 1989), and acting as an agonist and antagonist for GABAb receptors. Furthermore, 5‐AV suppresses convulsions in an epilepsy model (Dhaher *et al*. 2014). The decrease in 5‐AV in the cecum of sCSDS mice may also affect neuronal activities and behaviors related to depression.

## Perspectives for the Application of Rodent Models to Animal Science

Finally, I would like to describe the possible contributions that the study of rodent models of social defeat could make to animal science. The functional ingredients of animal products that prevent depression could be discovered by nutritional approaches using CSDS models. Furthermore, CSDS models will provide meaningful knowledge regarding animal welfare, particularly the management of social conflicts.

### Discovery of foods and nutrients for the prevention of depression

It is believed that rodent models of social defeat are suitable for discovering foods and nutritional factors that could potentially play a preventative role in depression. Given that there are antidepressant‐resistant patients, novel types of antidepressants such as ketamine, an NMDA receptor antagonist, are needed for clinical intervention (DeWilde *et al*. 2015). Recently, nutritional functionality has received attention with respect to the prevention of depression, because the intestinal environment and microbiota appear to be closely linked to various mental disorders (Lang *et al*. 2015; Stilling *et al*. 2016). Rodent models of social defeat will provide appropriate information regarding the type of nutrition suitable for preventing and treating depression. Unfortunately, it may be difficult to screen foods using social defeat models because some skills are needed for development and characterization of the defeat models. Simple and easy methods should therefore be developed for such screening using social defeat models.

Recently, we discovered that sCSDS mice delay nest‐building behavior (Otabi *et al*. 2016). Mouse nesting behavior is a complex, innate and goal‐directed behavior (Kinder 1927; Van de Weerd *et al*. 1997), and nest building requires attention related to particular areas of the frontal cortex and hippocampus (Kolb 1984; Carter *et al*. 2000; Deacon *et al*. 2002, 2003; Filali & Lalonde 2009). Because attention deficit is observed in depressive patients, nesting delay in sCSDS mice may represent one of the symptoms of depression. The deficiency in nest building behavior has been identified in various mouse mental illness models, including that of schizophrenia (Takao *et al*. 2013), whereas sCSDS mice can finally complete nests (Otabi *et al*. 2016). Similar symptoms have also been observed in a mouse model of Down's syndrome, and the symptoms of this delay in nest building were improved by antagonists of serotonin (5‐HT) receptor 2A (Heller *et al*. 2014). Otabi *et al*. (2017) reported that acute social defeat stress also delays nest building and that this delay is partially rescued by a 5‐HT2A receptor antagonist. Therefore, a paradigm that consists of acute social defeat stress and an evaluation of nest quality will be a convenient tool for screening functional foods and reagents that could potentially contribute to the prevention and treatment of depression. Furthermore, in the future, automatic analysis systems for the evaluation of nest quality should be developed for efficient screening (Okayama *et al*. 2015; Goto *et al*. 2016b).

### Application to animal welfare

Group‐housed livestock are constantly affected by social stress and conflict. Because the social ranking in group‐housed pigs affects growth performance (Desire *et al*. 2015), it is very important for livestock farmers to successfully manage social conflict and hierarchy in livestock. Comprehensive studies on nutritional and metabolic features in rodent models of social defeat will promote the development of feed additives that could reduce the distress effects attributable to social stress and increase resilience in livestock. In this regard, a recent publication has described that feedstuffs for cattle affect not only meat quality but also animal welfare (Carrillo *et al*. 2016), and that blood cortisol levels strongly indicate that grass‐fed animals may experience less stress than grain‐fed individuals (Carrillo *et al*. 2016). Although the quality of feed has a profound effect on livestock welfare, it remains difficult to conduct comprehensive and reproducible studies on this issue using livestock. We have found that feed purity influences stress resilience in mice (Goto *et al*. 2016a,b); therefore, we believe that rodent models provide relevant information relating to feeding management under stressful conditions. Moreover, zoo animals are exposed to various stresses, and abnormal behaviors such as pacing are frequently observed (Koyama *et al*. 2012). In addition to environmental enrichment, efforts should be made to discover the feeding conditions that could suppress such abnormal behaviors in zoo animals.

### Conclusion

Recent studies have revealed that observations on socially defeated rodents can potentially contribute not only to research on depression but also to various studies in animal science. This is because there probably exists a common mechanism for coping with stress in any social group of animals, including humans, experimental animals, livestock and zoo animals. Animal science researchers are expected to utilize and study the social defeat model from various aspects, which will promote a global understanding of the effects of social stress on animal nature.

## References

[asj12809-bib-0001] Altamura C , Maes M , Dai J , Meltzer HY . 1995 Plasma concentrations of excitatory amino acids, serine, glycine, taurine and histidine in major depression. European Neuropsychopharmacology 5(Suppl), 71–75. 8775762 10.1016/0924-977x(95)00033-l

[asj12809-bib-0002] American Psychiatric Association . 2013 Diagnostic and Statistical Manual of Mental Disorders (DSM‐5®), 5th edn American Psychiatric Association, Arlington, VA.

[asj12809-bib-0003] Aoki‐Yoshida A , Aoki R , Moriya N , Goto T , Kubota Y , Toyoda A , *et al* 2016 Omics studies of the murine intestinal ecosystem exposed to subchronic and mild social defeat stress. Journal of Proteome Research 15, 3126–3138. 27482843 10.1021/acs.jproteome.6b00262

[asj12809-bib-0004] Bagot RC , Parise EM , Peña CJ , Zhang HX , Maze I , Chaudhury D , *et al* 2015 Ventral hippocampal afferents to the nucleus accumbens regulate susceptibility to depression. Nature Communications 6, 7062. 10.1038/ncomms8062 PMC4430111 25952660

[asj12809-bib-0005] Carrillo JA , He Y , Li Y , Liu J , Erdman RA , Sonstegard TS , *et al* 2016 Integrated metabolomic and transcriptome analyses reveal finishing forage affects metabolic pathways related to beef quality and animal welfare. Scientific Reports 6, 25948. 27185157 10.1038/srep25948 PMC4869019

[asj12809-bib-0006] Carter PA , Swallow JG , Davis SJ , Garland T . 2000 Nesting behavior of house mice (*Mus domesticus*) selected for increased wheel‐running activity. Behavior Genetics 30, 85–94. 10979598 10.1023/a:1001967019229

[asj12809-bib-0007] Cryan JF , Holmes A . 2005 The ascent of mouse: advances in modelling human depression and anxiety. Nature Reviews Drug Discovery 4, 775–790. 16138108 10.1038/nrd1825

[asj12809-bib-0008] Cryan JF , Mombereau C . 2004 In search of a depressed mouse: utility of models for studying depression‐related behavior in genetically modified mice. Molecular Psychiatry 9, 326–357. 14743184 10.1038/sj.mp.4001457

[asj12809-bib-0009] Deacon RM , Croucher A , Rawlins JN . 2002 Hippocampal cytotoxic lesion effects on species‐typical behaviours in mice. Behavioural Brain Research 132, 203–213. 11997150 10.1016/s0166-4328(01)00401-6

[asj12809-bib-0010] Deacon RM , Penny C , Rawlins JN . 2003 Effects of medial prefrontal cortex cytotoxic lesions in mice. Behavioural Brain Research 139, 139–155. 12642185 10.1016/s0166-4328(02)00225-5

[asj12809-bib-0011] Desire S , Turner SP , D'Eath RB , Doeschl‐Wilson AB , Lewis CR , Roehe R . 2015 Genetic associations of short‐ and long‐term aggressiveness identified by skin lesion with growth, feed efficiency, and carcass characteristics in growing pigs. Journal of Animal Science 93, 3303–3312. 26439999 10.2527/jas.2014-8823

[asj12809-bib-0012] DeWilde KE , Levitch CF , Murrough JW , Mathew SJ , Iosifescu DV . 2015 The promise of ketamine for treatment‐resistant depression: current evidence and future directions. Annals of the New York Academy of Sciences 1345, 47–58. 25649308 10.1111/nyas.12646 PMC4447578

[asj12809-bib-0013] Dhaher R , Damisah EC , Wang H , Gruenbaum SE , Ong C , Zaveri HP , *et al* 2014 5‐aminovaleric acid suppresses the development of severe seizures in the methionine sulfoximine model of mesial temporal lobe epilepsy. Neurobiology of Disease 67, 18–23. 24632421 10.1016/j.nbd.2014.03.006 PMC4035438

[asj12809-bib-0014] Filali M , Lalonde R . 2009 Age‐related cognitive decline and nesting behavior in an APPswe/PS1 bigenic model of Alzheimer's disease. Brain Research 1292, 93–99. 19643098 10.1016/j.brainres.2009.07.066

[asj12809-bib-0015] Fritsche E , Humm A , Huber R . 1999 The ligand‐induced structural changes of human l‐Arginine: glycine amidinotransferase. A mutational and crystallographic study. The Journal of Biological Chemistry 274, 3026–3032. 9915841 10.1074/jbc.274.5.3026

[asj12809-bib-0016] Golden SA , Covington HE , Berton O , Russo SJ . 2011 A standardized protocol for repeated social defeat stress in mice. Nature Protocols 6, 1183–1191. 21799487 10.1038/nprot.2011.361 PMC3220278

[asj12809-bib-0017] Golden SA , Christoffel DJ , Heshmati M , Hodes GE , Magida J , Davis K , *et al* 2013 Epigenetic regulation of RAC1 induces synaptic remodeling in stress disorders and depression. Nature Medicine 19, 337–344. 10.1038/nm.3090 PMC3594624 23416703

[asj12809-bib-0018] Goto T , Toyoda A . 2015a A mouse model of subchronic and mild social defeat stress for understanding stress‐induced behavioral and physiological deficits. Journal of Visualized Experiments 105, e52973. 10.3791/52973 PMC4692742 26650680

[asj12809-bib-0019] Goto T , Kubota Y , Tanaka Y , Iio W , Moriya N , Toyoda A . 2014 Subchronic and mild social defeat stress accelerates food intake and body weight gain with polydipsia‐like features in mice. Behavioural Brain Research 270, 339–348. 24875770 10.1016/j.bbr.2014.05.040

[asj12809-bib-0020] Goto T , Kubota Y , Toyoda A . 2015b Plasma and liver metabolic profiles in mice subjected to subchronic and mild social defeat stress. Journal of Proteome Research 14, 1025–1032. 25437455 10.1021/pr501044k

[asj12809-bib-0021] Goto T , Kubota Y , Toyoda A . 2016a Effects of diet quality on vulnerability to mild subchronic social defeat stress in mice. Nutritional Neuroscience 19, 284–289. 25839213 10.1179/1476830515Y.0000000017

[asj12809-bib-0022] Goto T , Tomonaga S , Okayama T , Toyoda A . 2016b Murine depression model and its potential applications for discovering foods and farm products with antidepressant‐like effects. Frontiers in Neuroscience 10, 72. 26973450 10.3389/fnins.2016.00072 PMC4771721

[asj12809-bib-0023] Grandin T , Shivley C . 2015 How farm animals react and perceive stressful situations such as handling, restraint, and transport. Animals (Basel) 5, 1233–1251. 26633523 10.3390/ani5040409 PMC4693213

[asj12809-bib-0024] Heller HC , Salehi A , Chuluun B , Das D , Lin B , Moghadam S , *et al* 2014 Nest building is impaired in the Ts65Dn mouse model of Down syndrome and rescued by blocking 5HT2a receptors. Neurobiology of Learning and Memory 116, 162–171. 25463650 10.1016/j.nlm.2014.10.002

[asj12809-bib-0025] Hodes GE , Pfau ML , Leboeuf M , Golden SA , Christoffel DJ , Bregman D , *et al* 2014 Individual differences in the peripheral immune system promote resilience versus susceptibility to social stress. Proceedings of the National Academy of Sciences of the United States of America 111, 16136–13141. 25331895 10.1073/pnas.1415191111 PMC4234602

[asj12809-bib-0026] Iio W , Matsukawa N , Tsukahara T , Kohari D , Toyoda A . 2011 Effects of chronic social defeat stress on MAP kinase cascade. Neuroscience Letters 504, 281–284. 21970968 10.1016/j.neulet.2011.09.047

[asj12809-bib-0027] Iio W , Tokutake Y , Matsukawa N , Tsukahara T , Chohnan S , Toyoda A . 2012a Anorexic behavior and elevation of hypothalamic malonyl‐CoA in socially defeated rats. Biochemical and Biophysical Research Communications 421, 301–304. 22503976 10.1016/j.bbrc.2012.04.004

[asj12809-bib-0028] Iio W , Matsukawa N , Tsukahara T , Toyoda A . 2012b The effects of oral taurine administration on behavior and hippocampal signal transduction in rats. Amino Acids 43, 2037–2046. 22526240 10.1007/s00726-012-1282-2

[asj12809-bib-0029] Iio W , Takagi H , Ogawa Y , Tsukahara T , Chohnan S , Toyoda A . 2014 Effects of chronic social defeat stress on peripheral leptin and its hypothalamic actions. BMC Neuroscience 15, 72. 24906408 10.1186/1471-2202-15-72 PMC4059170

[asj12809-bib-0030] Iñiguez SD , Riggs LM , Nieto SJ , Dayrit G , Zamora NN , Shawhan KL , *et al* 2014 Social defeat stress induces a depression‐like phenotype in adolescent male c57BL/6 mice. Stress 17, 247–255. 24689732 10.3109/10253890.2014.910650 PMC5534169

[asj12809-bib-0031] Kasahara T , Takata A , Kato TM , Kubota‐Sakashita M , Sawada T , Kakita A , *et al* 2016 Depression‐like episodes in mice harboring mtDNA deletions in paraventricular thalamus. Molecular Psychiatry 21, 39–48. 26481320 10.1038/mp.2015.156 PMC5414076

[asj12809-bib-0032] Kinder EF . 1927 A study of the nest‐building activity of the albino rat. Journal of Experimental Zoology 47, 117–161.

[asj12809-bib-0033] Kolb B . 1984 Functions of the frontal cortex of the rat: a comparative review. Brain Research 320, 65–98. 6440660 10.1016/0165-0173(84)90018-3

[asj12809-bib-0034] Koyama N , Ueno Y , Eguchi Y , Uetake K , Tanaka T . 2012 Effects of daily management changes on behavioral patterns of a solitary female African elephant (*Loxodonta africana*) in a zoo. Animal Science Journal 83, 562–570. 22776795 10.1111/j.1740-0929.2011.00992.x

[asj12809-bib-0035] Krishnan V , Han MH , Graham DL , Berton O , Renthal W , Russo SJ , *et al* 2007 Molecular adaptations underlying susceptibility and resistance to social defeat in brain reward regions. Cell 131, 391–404. 17956738 10.1016/j.cell.2007.09.018

[asj12809-bib-0036] Lane MD , Wolfgang M , Cha SH , Dai Y . 2008 Regulation of food intake and energy expenditure by hypothalamic malonyl‐CoA. International Journal of Obesity (London) 32(Suppl 4), S49–S54. 10.1038/ijo.2008.123 18719599

[asj12809-bib-0037] Lang UE , Beglinger C , Schweinfurth N , Walter M , Borgwardt S . 2015 Nutritional aspects of depression. Cellular Physiology and Biochemistry 37, 1029–1043. 26402520 10.1159/000430229

[asj12809-bib-0038] Lutter M , Nestler EJ . 2009 Homeostatic and hedonic signals interact in the regulation of food intake. Journal of Nutrition 139, 629–632. 19176746 10.3945/jn.108.097618 PMC2714382

[asj12809-bib-0039] Marcinkiewicz J , Kontny E . 2014 Taurine and inflammatory diseases. Amino Acids 46, 7–20. 22810731 10.1007/s00726-012-1361-4 PMC3894431

[asj12809-bib-0040] McKim DB , Niraula A , Tarr AJ , Wohleb ES , Sheridan JF , Godbout JP . 2016 Neuroinflammatory dynamics underlie memory impairments after repeated social defeat. Journal of Neuroscience 36, 2590–2604. 26937001 10.1523/JNEUROSCI.2394-15.2016 PMC4879207

[asj12809-bib-0041] Mechawar N , Savitz J . 2016 Neuropathology of mood disorders: do we see the stigmata of inflammation? Translational Psychiatry 6, e946. 27824355 10.1038/tp.2016.212 PMC5314124

[asj12809-bib-0042] Miczek KA . 1979 A new test for aggression in rats without aversive stimulation: differential effects of d‐amphetamine and cocaine. Psychopharmacology – Berlin 60, 253–259. 10.1007/BF00426664 108702

[asj12809-bib-0043] Murakami T , Furuse M . 2010 The impact of taurine‐ and beta‐alanine‐supplemented diets on behavioral and neurochemical parameters in mice: antidepressant versus anxiolytic‐like effects. Amino Acids 39, 427–434. 20099004 10.1007/s00726-009-0458-x

[asj12809-bib-0044] Nanavati SM , Silverman RB . 1989 Design of potential anticonvulsant agents: mechanistic classification of GABA aminotransferase inactivators. Journal of Medicinal Chemistry 32, 2413–2421. 2681782 10.1021/jm00131a001

[asj12809-bib-0045] Nestler EJ , Hyman SE . 2010 Animal models of neuropsychiatric disorders. Nature Neuroscience 13, 1161–1169. 20877280 10.1038/nn.2647 PMC3750731

[asj12809-bib-0046] Okayama T , Goto T , Toyoda A . 2015 Assessing nest‐building behavior of mice using a 3D depth camera. Journal of Neuroscience Methods 251, 151–157. 26051553 10.1016/j.jneumeth.2015.05.019

[asj12809-bib-0047] Oliveira RF , Simões JM , Teles MC , Oliveira CR , Becker JD , Lopes JS . 2016 Assessment of fight outcome is needed to activate socially driven transcriptional changes in the zebrafish brain. Proceedings of the National Academy of Sciences of the United States of America 113, E654–E661. 26787876 10.1073/pnas.1514292113 PMC4747755

[asj12809-bib-0048] Otabi H , Goto T , Okayama T , Kohari D , Toyoda A . 2016 Subchronic and mild social defeat stress alter mouse nest building behavior. Behavioural Processes 122, 21–25. 26524409 10.1016/j.beproc.2015.10.018

[asj12809-bib-0049] Otabi H , Goto T , Okayama T , Kohari D , Toyoda A . 2017 The acute social defeat stress and nest‐building test paradigm: a potential new method to screen drugs for depressive‐like symptoms. Behavioural Processes 135, 71–75. 27939810 10.1016/j.beproc.2016.12.003

[asj12809-bib-0050] Penn JK , Zito MF , Kravitz EA . 2010 A single social defeat reduces aggression in a highly aggressive strain of Drosophila. Proceedings of the National Academy of Sciences of the United States of America 107, 12682–12686. 20616023 10.1073/pnas.1007016107 PMC2906583

[asj12809-bib-0051] Porsolt RD , Le Pichon M , Jalfre M . 1977 Depression: a new animal model sensitive to antidepressant treatments. Nature 266, 730–732. 559941 10.1038/266730a0

[asj12809-bib-0052] Potegal M , Huhman K , Moore T , Meyerhoff J . 1993 Conditioned defeat in the Syrian golden hamster (*Mesocricetus auratus*). Behavioral and Neural Biology 60, 93–102. 8117243 10.1016/0163-1047(93)90159-f

[asj12809-bib-0053] Rillich J , Stevenson PA . 2014 A fighter's comeback: dopamine is necessary for recovery of aggression after social defeat in crickets. Hormones and Behavior 66, 696–704. 25268421 10.1016/j.yhbeh.2014.09.012

[asj12809-bib-0054] Ruis MA , de Groot J , te Brake JH , Dinand Ekkel E , van de Burgwal JA , Erkens JH , *et al* 2001 Behavioural and physiological consequences of acute social defeat in growing gilts: effects of the social environment. Applied Animal Behaviour Science 70, 201–225. 11118662 10.1016/s0168-1591(00)00150-7

[asj12809-bib-0055] Rygula R , Abumaria N , Flügge G , Fuchs E , Rüther E , Havemann‐Reinecke U . 2005 Anhedonia and motivational deficits in rats: impact of chronic social stress. Behavioural Brain Research 162, 127–134. 15922073 10.1016/j.bbr.2005.03.009

[asj12809-bib-0056] Savignac HM , Finger BC , Pizzo RC , O'Leary OF , Dinan TG , Cryan JF . 2011 Increased sensitivity to the effects of chronic social defeat stress in an innately anxious mouse strain. Neuroscience 192, 524–536. 21635938 10.1016/j.neuroscience.2011.04.054

[asj12809-bib-0057] Schmidt M , Oitzl MS , Levine S , de Kloet ER . 2002 The HPA system during the postnatal development of CD1 mice, the effects of maternal deprivation. Brain Research Developmental Brain Research 139, 39–49. 12414092 10.1016/s0165-3806(02)00519-9

[asj12809-bib-0058] Schubring SR , Fleischer W , Lin JS , Haas HL , Sergeeva OA . 2012 The bile steroid chenodeoxycholate is a potent antagonist at NMDA and GABA(A) receptors. Neuroscience Letters 506, 322–326. 22155097 10.1016/j.neulet.2011.11.036

[asj12809-bib-0059] Selye H . 1956 The Stress of Life. McGraw‐Hill, New York, USA.

[asj12809-bib-0060] Shen Y , Connor TJ , Nolan Y , Kelly JP , Leonard BE . 1999 Differential effect of chronic antidepressant treatments on lipopolysaccharide‐induced depressive‐like behavioural symptoms in the rat. Life Science 65, 1773–1786. 10.1016/s0024-3205(99)00430-0 10576557

[asj12809-bib-0061] Sial OK , Warren BL , Alcantara LF , Parise EM , Bolaños‐Guzmán CA . 2016 Vicarious social defeat stress: bridging the gap between physical and emotional stress. Journal of Neuroscience Methods 258, 94–103. 26545443 10.1016/j.jneumeth.2015.10.012 PMC4691556

[asj12809-bib-0062] Solomon MB . 2017 Evaluating social defeat as a model for psychopathology in adult female rodents. Journal of Neuroscience Research 95, 763–776. 27870445 10.1002/jnr.23971

[asj12809-bib-0063] Steru L , Chermat R , Thierry B , Simon P . 1985 The tail suspension test: a new method for screening antidepressants in mice. Psychopharmacology – Berlin 85, 367–370. 10.1007/BF00428203 3923523

[asj12809-bib-0064] Stilling RM , van de Wouw M , Clarke G , Stanton C , Dinan TG , Cryan JF . 2016 The neuropharmacology of butyrate: the bread and butter of the microbiota‐gut‐brain axis? Neurochemistry International 99, 110–132. 27346602 10.1016/j.neuint.2016.06.011

[asj12809-bib-0065] Takao K , Kobayashi K , Hagihara H , Ohira K , Shoji H , Hattori S , *et al* 2013 Deficiency of schnurri‐2, an MHC enhancer binding protein, induces mild chronic inflammation in the brain and confers molecular, neuronal, and behavioral phenotypes related to schizophrenia. Neuropsychopharmacology 38, 1409–1425. 23389689 10.1038/npp.2013.38 PMC3682135

[asj12809-bib-0066] Tasaka J , Sakai S , Tosaka T , Yoshihama I . 1989 Glial uptake system of GABA distinct from that of taurine in the bullfrog sympathetic ganglia. Neurochemical Research 14, 271–277. 2786163 10.1007/BF00971323

[asj12809-bib-0067] Van de Weerd HA , Van Loo PL , Van Zutphen LF , Koolhaas JM , Baumans V . 1997 Preferences for nesting material as environmental enrichment for laboratory mice. Laboratory Animals 31, 133–143. 9175010 10.1258/002367797780600152

[asj12809-bib-0068] Weissman MM , Bland RC , Canino GJ , Faravelli C , Greenwald S , Hwu HG , *et al* 1996 Cross‐national epidemiology of major depression and bipolar disorder. Journal of the American Medical Association 276, 293–299. 8656541

[asj12809-bib-0069] Willner P . 2005 Chronic mild stress (CMS) revisited: consistency and behavioural‐neurobiological concordance in the effects of CMS. Neuropsychobiology 52, 90–110. 16037678 10.1159/000087097

[asj12809-bib-0070] Yao W , Zhang JC , Ishima T , Dong C , Yang C , Ren Q , *et al* 2016 Role of Keap1‐Nrf2 signaling in depression and dietary intake of glucoraphanin confers stress resilience in mice. Scientific Reports 6, 30659. 27470577 10.1038/srep30659 PMC4965765

